# Comparison of endothelial cell loss in diabetic patients after conventional phacoemulsification and femtosecond laser-assisted cataract surgery

**DOI:** 10.1186/s12886-023-02923-3

**Published:** 2023-04-26

**Authors:** João Carlos Gonçalves Cruz, Celso Busnelo Moreno, Paula Virginia Brom dos Santos Soares, Bernardo Kaplan Moscovici, Guilherme Novoa Colombo-Barboza, Luiz Roberto Colombo-Barboza, Marcello Novoa Colombo-Barboza

**Affiliations:** 1 Hospital Oftalmológico Visão Laser, Avenida Conselheiro Nébias, 355, Santos, São Paulo, 11015-003 Brazil; 2 Department of Ophthalmology and Visual Sciences, Universidade Federal de São Paulo (EPM/UNIFESP), São Paulo, Brazil; 3 Universidade Metropolitana de Santos (UNIMES), Santos, Brazil; 4 Faculdade de Ciências Médicas de Santos (UNILUS), Santos, Brazil

**Keywords:** Cataract, Cataract extraction, Phacoemulsification, Type 2 diabetes mellitus, Corneal endothelium

## Abstract

**Purpose:**

This study aims to comparatively evaluate the morphological changes of the cornea after phacoemulsification (PHACO) and femtosecond laser-assisted cataract surgery (FLACS) without intercurrences in patients with type 2 diabetes mellitus.

**Methods:**

A total of 95 diabetic patients with moderate cataracts (N2 + and N3+), 47 undergoing PHACO and 48 undergoing FLACS, were selected randomly for the study. Surgeries were performed by a single surgeon between July 2021 and December 2021. Cumulative dissipated energy (CDE) and total balanced saline solution (BSS) data were obtained at the end of each surgery. Changes in corneal endothelial cell density (ECD) and central corneal thickness (CCT) at three months postoperatively were investigated.

**Results:**

After three months, evidence is lacking between groups in the CCT measures; the difference was neither statistically nor clinically relevant. However, for ECD, a significant and clinically significant difference was found; if all patients were treated with laser, the mean ECD would be 423.55 greater (RSE: 86.09; p-value < 0.001; 95% CI: 254.81–592.29) than the ECD potential means of 1656.423 among the conventional group (RSE: 74.90; p-value < 0.001; 95% CI: 1509.62–1803.23).

**Conclusions:**

Diabetic patients under treatment with moderate cataracts may predispose themselves to a more significant loss of endothelial cells after conventional phacoemulsification than femtosecond laser-assisted cataract surgery.

**Trial registration:**

It was registered at The Brazilian Registry of Clinical Trials (ReBEC) with the code RBR-6d8whb5 (UTN code: U1111-1277-6020) on 17/05/2022.

## Introduction

Cataract surgery is one of the most performed surgeries worldwide [1]. Currently, phacoemulsification is the standard surgical technique for cataract extraction used in the developed world [2]. With the advancement of surgical equipment, the femtosecond laser appears to minimize the effect of phacoemulsification energy and its complications.

The use of femtosecond (FL) laser has been consolidated for a long time in corneal surgery; however, it is relatively recent in cataract surgery. This technology uses ultrashort pulses of infrared light to disrupt tissue precisely, minimizing collateral damage to adjacent tissues [3]. Femtosecond laser-guided cataract surgery (FLACS) has shown benefits in specific surgical parameters, including decreased energy dissipation, improved capsulotomy circularity, reduced postoperative corneal edema, and loss of corneal endothelial cells [4,5].

The safety of phacoemulsification has been validated recently, yet surgeons must be aware of the possible risk factors for complicated eyes. Studies have investigated the relationship between elevated plasma glucose concentrations in diabetes mellitus (DM) and the morphological and functional properties of the cornea. The hyperglycemia found in diabetes mellitus is responsible for several biochemical and structural abnormalities of the cornea, including the accumulation of advanced glycosylated end products, which can lead to a cell adhesion defect; [6] increased expression of metalloproteinase, which damages the basement membrane and limits cell migration [5], and inhibition of endothelial Na+/k + ATPase activity, resulting in corneal edema and reduced transparency [7]. Because of these, diabetic corneas are possibly more vulnerable to stress and trauma caused by cataract surgery.

Studies have investigated the effect of conventional phacoemulsification among diabetic patients, some showing more significant loss of endothelial cells in the diabetic group than those in the control group [8,9]. In contrast, other studies showed no significant difference between the groups [10–13]. When performing FLACS, the work by Kang et al. showed that no statistically significant difference in the loss of endothelial cells was found between diabetic and control patients; however, this was a retrospective study with only 31 diabetic patients [14].

This study aimed to evaluate the cornea’s morphological changes after conventional phacoemulsification (PHACO) and FLACS surgery without intercurrences in patients with type 2 diabetes mellitus.

## Materials and methods

### Study population

This study was conducted at the Department of Ophthalmology, Hospital Oftalmológico Visão Laser, Santos, São Paulo, Brazil, between July 2021 and December 2021. The study was approved by the Ethical Committee, Universidade Metropolitana de Santos, Brazil, and all aspects of the Declaration of Helsinki were observed.

### Inclusion and exclusion criteria

A total of 95 diabetic patients, aged 50 to 85, with moderate cataracts (N2 + and N3+), 47 undergoing PHACO and 48 undergoing FLACS, were selected randomly for the study. All patients were diagnosed with type 2 diabetes mellitus under clinical treatment (glycated hemoglobin < 7.0 being the criterion used) who presented senile cataract and low visual acuity, which required surgical intervention and were willing to participate in the study.

Exclusion criteria were the same in both groups: moderate to severe diabetic retinopathy, diabetic macular edema, traumatic cataract, mature and hypermature cataract, LOCS III nuclear cataract graduation above 4, pseudoexfoliation syndrome, significant corneal opacity, glaucoma, ocular hypertension, uveitis, vitreoretinal pathologies, previous intraocular surgery, intraoperative complications during cataract surgery, collagen vascular disease, any active eye disease, high myopia, high hyperopia (axial length < 15 mm), age of < 50 years, preoperative cell count of < 1,000 per square millimeter or preoperative anterior chamber depth of < 2.5 mm, iris neovascularization, or other abnormalities that could significantly impair endothelial cells regardless of surgery.

### Cataract graduation

The Lens Opacity Classification System III (LOCS III) was used to grade the cataract, and the same ophthalmologist performed the assessment using a slit lamp with maximum illumination without a light filter and only on patients with nuclear density 2 + and 3 + for the study [15].

### Preoperative exams

First, a detailed medical history was obtained, and a single ophthalmologist performed a complete physical examination to properly recruit participants for the study. Examinations included best-corrected visual acuity using the Snellen chart, refraction, biomicroscopy, Goldman tonometry, gonioscopy, and indirect ophthalmoscopy under drug-induced mydriasis for posterior segment assessment. The IOL Master® 500 (Carl Zeiss Meditec, Jena, Germany) was used to calculate intraocular lens (IOL). Corneal endothelial morphological properties were evaluated using the Topcon® SP-3000P noncontact specular microscope. To exclude the presence of any macular edema, optical coherence tomography of the macula was performed with the Heidelberg Spectralis (Heidelberg Engineering, Heidelberg, Germany).

### Surgical procedure

The same surgeon performed all surgeries.

The FLACS group performed pretreatment using the LenSx laser (Alcon Laboratories, Fort Worth, TX, USA). After successfully coupling the laser-patient interface, spectral domain optical coherence tomography imaging of the anterior segment was performed. Further, laser treatment was initiated and included the creation of a 5.5-mm capsulotomy and lens fragmentation (LenSx pattern: concentric cylinders and segmental cuts). Once pretreatment was completed, a 1.2-mm one-plane paracentesis and a 2.75-mm two-plane main incision were made using disposable keratome knives, following phacoemulsification, performed using the Centurion System (Alcon Laboratories, Fort Worth, TX, USA). A dispersive viscoelastic was injected into the anterior chamber to protect the endothelium. The anterior capsule was removed using utrata forceps and hydrodissection followed. Surgery was then completed with the standard phacoemulsification procedure. The remaining cortex was removed through coaxial irrigation/aspiration, followed by IOL implantation. Stromal hydration was applied to close the corneal incisions at the end of the procedure. A combined steroid-antibiotic ointment was placed on the eye, and then the eye was covered with a clear acrylic shield.

In the PHACO group, a 1.2-mm one-plane paracentesis and a 2.75-mm two-plane main incision were made using disposable keratome knives. Continuous curvilinear capsulorrhexis was completed using utrata forceps through the main incision. Surgery was then completed with the standard phacoemulsification procedure with a phacochop technique. The remaining steps were similar to those described in the FLACS group.

### Intraoperative data collection

A single nurse recorded the intraoperative measurements. Accumulated energy dissipated (CDE) data and total balanced saline solution were obtained at the end of each surgery. The Centurion System (Alcon Laboratories, Fort Worth, TX, USA) uses the CDE as a value for phacoemulsification energy, calculated as (longitudinal time × average longitudinal power) + (torsional time × 0.4 × average torsional amplitude) [16].

### Postoperative data collection

Two outcome measures were considered for analyzing endothelial cells after surgery: corneal endothelial cell density (ECD) and central corneal thickness (CCT). The examiner was blinded, so he did not know to which group each patient belonged.

CCT and ECD were measured using a Topcon® SP-3000P noncontact specular microscope (Topcon, Tokyo, Japan). The central method was used for cell counting. Three readings were taken and an average was taken as a result. Extreme variations in values were discarded [17].

The other variables shown in specular microscopy were also analyzed, such as number of cells, average size, standard deviation and the coefficient of variation of the size, and the hexagonality.

### Sample calculation

The following input parameters under a family, two-tailed t-test, aiming to test the difference between means between two independent groups, were used to estimate the sample size for this quasi-experiment: the probability of alpha error of 0.05, power of 0.80, minimum detectable effect size Cohen’s d of 0.6 (mean effect size), and an allocation ratio of 1:1. Under such parameters, a sample size of at least 45 FLACS vs. 45 PHACO would at least be needed.

### Statistical analysis

For continuous measurements, descriptive statistics were presented in terms of minimum, maximum, mean, and standard deviation. For categorical measurements, proportions were used. To test for group difference in continuous baseline measures, the t-test or the Mann–Whitney U test was used depending on the normal distribution of measures within each group and assumptions of equal variances; when the t-test was used, the effect size was reported in Cohen’s d, where 0.2, 0.5, and 0.8 represent small, medium, and large differences in effect sizes, respectively. For the Mann–Whitney U tests, effect sizes are given by biserial correlation classification. The chi-square test was used to test for the differences in proportion among sex, laterality, and type of cataract.

The effect of the quasi-experiment design was assessed using the treatment effect via regression adjustment estimator. Two indicators of the effect were calculated: average treatment effect (ATE) and the potential-outcome means for each treatment level.

All the models were run using STATA (version 14). The adopted statistical significance threshold was 0.05.

For all specular microscope variables we used the Mann-Whitney test for analysis. This comparison was made both in the pre and post. But we also compared the groups for gain, which is the simple mathematical difference of post-pre (when positive, it means an increase, but when negative, it means a reduction).

## Results

Tables 1 and 2 describe the studied population and pre-and postoperative data. For quantitative variables, we described mean, +- standard deviation (SD), maximum and minimum value; for qualitative variables, include absolute and relative frequencies.

**Table 1:** Descripte statistics for categorical measures per group

	Laser	Conventional	Chi-square
Sex (Male)	59.6%	25%	0.001
Cataract N2	59.6%	62,5%	0.770
Laterality (Right)	53.2%	43.8%	0.357

**Table 2:** Descriptive statistics for baseline continuous features per group

	Laser (N = 47)				Conventional (N = 48)					
	Minimum	Maximum	Mean	Std. Deviation	Minimum	Maximum	Mean	Std. Deviation	p-value	Cohen’s d
Age	57.0	85.0	71.4	6.4	50.0	84.0	69.9	8.2	0.297	-0.215
VA LogMar pre	0.3	0.4	0.3	0.1	0.3	0.4	0.3	0.1	0.911	0.023
VA LogMar post	0.0	0.1	0.0	0.1	0.0	0.1	0.0	0.1	0.768	-0.061
CDE	5.2	7.0	5.9	0.5	5.2	7.0	6.1	0.6	0.131	0.313
BSS	72.0	89.0	88.2	5.1	72.0	89.0	80.8	6.0	0.027	0.462
Pre-CCT	423.0	588.0	513.5	43.4	460.0	663.0	518.4	35.0	0.991	0.014
Pre-ECD	1798.0	3123.0	2416.5	326.9	1378.0	3219.0	2394.6	431.0	0.781	-0.057

VA: visual acuity; CDE: cumulative dissipated energy; BSS: balanced salt solution; CCT: central corneal thickness; ECD: endothelial cell density.

As demonstrated in these tables, the groups show differences in sex (the FLACS group has a higher proportion of men), and BSS was lower within the same group (close to an average effect size, Cohen’s d = 0.462, p-value = 0.027). All the other measurements showed that both groups are well balanced regarding demographic and clinical characteristics (small Cohen’s d effect sizes close to 0.2), i.e., not statistically and clinically relevant. Since the study is observational, to estimate the effects of PHACO vs. FLACS, considering the average consumption of BSS was necessary to match the groups. Gender was not used in the modeling because, regardless of its statistical significance, it is not clinically relevant, given that our results are anatomical measurements.

After three months, evidence was lacking between the groups in the CCT; the difference was neither statistically nor clinically relevant. If all patients were treated with laser, the mean CCT would be − 0.08 lower (robust standard error [RSE] = 8.64, p-value = 0.992, 95% confidence interval [CI] = − 17.03–16.86) on the CCT measure than the potential outcome means of 527.74 (RSE = 5.59, p-value < 0.001, 95% CI = 516.77–538.71). In other words, it is not statistically significant.

However, for ECD, a significant and clinically significant difference was found; if all patients were treated with laser, the mean ECD would be 423.55 greater (RSE: 86.09; p-value < 0.001; 95% CI: 254.81–592.29) than the ECD potential means of 1656.423 among the conventional arm (RSE: 74.90; p-value < 0.001; 95% CI: 1509.62–1803.23).

Table 3 presents corneal results after three months of intervention.

**Table 3:** Descriptive statistics for post intervention outcomes (left side) and treatment average effect adjusted by BSS (right side)

Descriptive post intervention						Effect adjusted for BSS		
Outcome after three months	Group	N	Mean	SD	SE	Average treatment Effect	Robust Std. Err	p-value
CCT	Conventional	48	528	35.555	5.1318			
	Laser	47	528.128	44.629	6.5098	-0.0844	8.64	0.992
ECD	Conventional	48	1628.479	521.641	75.292			
	Laser	47	2118.17	363.132	52.968	423.5548	86.09	< 0.001

CCT: central corneal thickness; ECD: endothelial cell density; N: sample

Finally, Table 4 compares the Conventional and Laser groups for the result of all specular microscope variables. We verify that in pre surgery there is no significant difference between the groups in all variables, that is, they are homogeneous in the initial evaluation in both groups. But in the post surgery we found a significant difference. The hexagonality was the only variable that showed difference in the pre and no difference in the post surgery.

**Table 4:** Comparison of specular microscope data between groups

			Average	Median	Standard deviation	N	CI	P-value
Cells	Pre	Conventional	95,3	100	19,8	48	5,6	0,491
		Laser	98,1	101	17,9	47	5,1	
	Post	Conventional	63,2	63	22,4	48	6,3	< 0,001
		Laser	83,1	86	18,8	47	5,4	
	Gain	Conventional	-32,1	-29	24,9	48	7,0	< 0,001
		Laser	-15,0	-11	12,7	47	3,6	
Average Size	Pre	Conventional	429,0	403	88,0	48	24,9	0,873
		Laser	421,2	406	60,3	47	17,2	
	Post	Conventional	660,6	534	271,7	48	76,9	0,001
		Laser	475,9	468	92,6	47	26,5	
	Gain	Conventional	231,6	128	246,3	48	69,7	< 0,001
		Laser	54,7	27	74,1	47	21,2	
SD	Pre	Conventional	158,8	142	41,7	48	11,8	0,809
		Laser	156,2	139	48,2	47	13,8	
	Post	Conventional	238,6	210	108,8	48	30,8	0,010
		Laser	182,3	166	55,3	47	15,8	
	Gain	Conventional	79,8	45	94,2	48	26,6	0,001
		Laser	26,1	16	41,3	47	11,8	
CV	Pre	Conventional	36,9	35	7,1	48	2,0	0,729
		Laser	36,8	35	9,1	47	2,6	
	Post	Conventional	37,7	35	12,3	48	3,5	0,014
		Laser	40,1	39	8,4	47	2,4	
	Gain	Conventional	0,79	0	9,45	48	2,67	0,008
		Laser	3,30	4	6,37	47	1,82	
Hexagonality	Pre	Conventional	51,7	51	9,3	48	2,6	0,010
		Laser	56,3	57	7,7	47	2,2	
	Post	Conventional	47,1	50	12,7	48	3,6	0,566
		Laser	49,4	50	8,2	47	2,4	
	Gain	Conventional	-4,65	-4	14,29	48	4,04	0,053
		Laser	-6,91	-6	5,23	47	1,50	

SD: standard deviation of the size; CV: coefficient of variation of the size; CI: confidence interval; N: sample.

## Discussion

This study evaluated the structural and endothelial outcomes of the cornea after uneventful PHACO and FLACS in type 2 diabetic patients. Although the loss of endothelial cells (ECL) and variations in corneal thickness after cataract surgery in diabetic subjects have been reported in previous studies, many of these studies compared DM and non-DM groups, in addition to evaluating a single surgical technique.

This study showed a statistically significant difference between the groups regarding the loss of corneal endothelial cells and the use of BSS. The FLACS group had less loss of endothelial cells and less use of BSS in surgery. Considering this difference in using BSS, an adjustment was applied to the conventional group. If treated with laser, a statistically significant difference would still be observed between the groups, favoring the laser group.

Diabetic corneas result in more remarkable morphological changes in the corneal endothelium and longer recovery time after surgery. Several studies have reported the risk of corneal endothelial damage in diabetic patients with a higher ECL after conventional cataract surgery than in nondiabetic patients. Diabetic endothelium was more metabolically stressed and had less functional reserve than normal corneal endothelium [8,9]. Accordingly, our study showed a mean preoperative ECD of 2394.6 and postoperatively of 1628,479 in the PHACO group.

FLACS requires less phacoemulsification energy because the laser fragments the core. As the ECL correlates with the energy used, FLACS reduces the ECL more than conventional surgery.^17^–^19^ Some studies have shown less endothelial cell loss in normal eyes in patients undergoing FLACS than in conventional surgery.^[20]–[21]^ Chen et al. performed a Meta-analysis of 25 randomized controlled trials and found no statistically significant difference between FLACS and PHACO in clinical outcomes at the long-term follow-up. The postoperative CCT was significantly lower in the FLACS group compared to the PHACO group at one day and one week. However, it was not statistically significant at three months. FLACS reduced the postoperative ECL compared to PHACO at three months [22]. However, there was no significant difference between the two groups at six months. Although our study compared diabetic patients in a 3-month follow-up, until this period, our results are similar to the non-diabetic population.

In the literature, only one study with FLACS has evaluated the loss of corneal endothelial cells among diabetic patients. Kang et al. studied FLACS in diabetic and nondiabetic patients and showed no significant difference between the two groups regarding corneal endothelial cell damage. However, this retrospective study with a small sample of patients showed a more significant number of grade 4 or white nuclear cataracts in the nondiabetic group, which could explain the result of the study [14]. Conversely, in our study, the laser group showed less loss of endothelial cells than the conventional group, suggesting more excellent protection in the group submitted to FLACS, as described in the literature.

Some studies have shown that irrigation volume was significantly associated with a postoperative reduction in ECD [5]. A significant difference was found in BSS volume used between the groups, lesser in the FLACS group, favoring this group. Despite a lesser volume of BSS used, a correction was made to show the impact of this difference in the conventional group if it was treated with laser. It was shown a lower cell loss, but still clinically and statistically significant.

The results of cell loss reflect on the variables measured in specular microscopy. The group submitted to conventional phacoemulsification showed greater loss of analyzed cells, greater mean size, greater standard deviation and coefficient of variation of cell size in comparison to the laser group. However, there was no statistical difference regarding hexagonality after surgery between groups.

Regarding CCT, understanding that a more significant loss of endothelial cells does not necessarily impact corneal thickness is essential, even in patients with pathologies including Fuchs, as evidenced by the work by Wei Danya et al. This study showed a better performance of FLACS concerning endothelial cell density. However, no statistically significant difference was observed in central corneal thickness compared to that in the control group [23]. In agreement, our study found no statistically significant difference between the groups at the end of 3 months in the CCT value, despite the loss of endothelial cells. Probably, for the patients who presented a more accentuated endothelial loss, the quantity and quality of cells were sufficient to maintain corneal homeostasis.

Our results did not show statistically significant difference in postoperative visual acuity between the two techniques over the follow-up period. These findings are consistent with the majority of published studies that have not found significant postoperative benefit of FLACS over conventional surgery [24,25].

The literature shows that femtosecond laser-assisted cataract surgery seems to cause less damage to the corneal endothelium because it uses less phacoemulsification energy [14,16]. Although our study demonstrated no difference in CDE between the groups, this could be explained by the same technique used in both groups and the grade of the cataracts, which didn’t require much energy.

This research excluded patients with more advanced stages of diabetic retinopathy, mature cataracts, and complicated surgeries; therefore, compared to conventional surgery, FLACS for hard nuclear cataract surgery has been shown to reduce corneal endothelial damage, reduce corneal endothelial loss from 19.96 to 7.85%, and reduce vision recovery time from 3 months to 1 month [26].

Until now, surgery remains the only effective method for treating cataracts. There is still much to learn about complicated eyes and predict possible complications, despite the many relevant meaningful previous results. As for limitation, our study follows up patients just for 3 months, any conclusions beyond this long could not be drawn.

This study showed that despite reasonable glycemic control, corneal endothelial cells from diabetic patients are fragile to the stress caused by cataract surgery. Therefore, other factors may contribute to the increase in this vulnerability. Moreover, morphometric parameters did not translate into functional deficits, as observed by nonsignificant changes in CCT. Even so, the corneal endothelium of diabetic patients shows significantly greater damage after PHACO than FLACS, which justifies a more careful approach during surgery or even considering FLACS for these patients.

In conclusion, diabetic patients under treatment with moderate cataracts may predispose themselves to more significant endothelial cell loss after PHACO than FLACS. Additionally, no significant difference was observed in the CDE between the groups, leading us to believe that it is not the best parameter to assess the delivery of ultrasound energy in surgery.

## Data Availability

The datasets used and/or analysed during the current study available from the corresponding author on reasonable request.
